# Radon Concentrations in Drinking Water in Beijing City, China and Contribution to Radiation Dose

**DOI:** 10.3390/ijerph111111121

**Published:** 2014-10-27

**Authors:** Yun-Yun Wu, Yong-Zhong Ma, Hong-Xing Cui, Jian-Xiang Liu, Ya-Ru Sun, Bing Shang, Xu Su

**Affiliations:** 1 Key Laboratory of Radiological Protection and Nuclear Emergency, National Institute for Radiological Protection, Chinese Centre for Disease Control and Prevention, Beijing 100088, China; E-Mails: wuyunyun-001@163.com (Y.-Y.W.); hxcuicn@163.com (H.-X.C.); jxliu@163.com (J.-X.L.); shangbing66@163.com (B.S.); 2 Institute for Radiological Protection, Beijing Centre for Disease Control and Prevention, Beijing 100013, China; E-Mails: myz0905@126.com (Y.-Z.M.); sunyr_cdc@163.com (Y.-R.S.)

**Keywords:** radon, drinking water, well water, public water, radiation dose

## Abstract

^222^Rn concentrations in drinking water samples from Beijing City, China, were determined based on a simple method for the continuous monitoring of radon using a radon-in-air monitor coupled to an air-water exchanger. A total of 89 water samples were sampled and analyzed for their ^222^Rn content. The observed radon levels ranged from detection limit up to 49 Bq/L. The calculated arithmetic and geometric means of radon concentrations in all measured samples were equal to 5.87 and 4.63 Bq/L, respectively. The average annual effective dose from ingestion of radon in drinking water was 2.78 μSv, and that of inhalation of water-borne radon was 28.5 μSv. It is concluded that it is not the ingestion of waterborne radon, but inhalation of the radon escaping from water that is a substantial part of the radiological hazard. Radon in water is a big concern for public health, especially for consumers who directly use well water with very high radon concentration.

## 1. Introduction

Radon (^222^Rn) is a naturally occurring, short-lived (half-life of 3.825 days) radioactive decay product of uranium, and is found in various concentrations in soil, air and in different types of water as a result of migration from rocks and soil in contact with the water [1]. The primary health effect of radon is lung cancer, resulting from inhalation of radon in indoor air. There is also evidence from epidemiology and modeling studies that ingestion of radon can cause stomach cancer [2].

Estimated population risks from radon in drinking water via the inhalation and ingestion routes, separately and combined, were determined by the National Research Council (NRC) [2]. It has estimated that 89% of the estimated cancer risk resulted from inhalation of the radon emitted from water, and 11% caused by ingestion pathway.

The United States Environmental Protection Agency (EPA) proposed in 1991 a maximum contamination level (MCL) for radon of 11 Bq/L in drinking water [3]. However, from practical reasons in 1999 EPA recommends also another alternative maximum contamination level (AMCL) of 148 Bq/L considering the contribution to radon concentration in indoor air from household usage of water [4]. The World Health Organization (WHO) guidelines for drinking water quality suggest that repeated measurements should be implemented if radon activity concentration in public drinking water supplies exceeds 100 Bq/L [5]. Similar approach has been proposed in the EU (European Union) commission recommendations: no remedial action should be required if the concentration of radon in drinking water is <100 Bq/L [6]. Therefore, seven European countries (Denmark, Finland, Germany, Greece, Ireland, Sweden and the Czech Republic) have set their own reference levels in the range 20–1000 Bq/L for radon in drinking water [7].However, at present there is no reference level for radon in drinking water in China.

Many studies have been done to measure radon in water from different places around the world due to radon health hazard [8,9,10,11,12,13]. The authors have carried out a survey of radon concentration in public water supplies and well waters in Beijing City, China. The aims of this study are to draw a general picture of the radon activity of drinking water in Beijing City and to evaluate doses to the population resulting from the ingestion and inhalation of radon from water. 73 water samples from public water supply and 16 well water samples were collected in the city and surroundings. As far as is known, this is the first detailed study to determine radon concentration in public water supplies and well waters separately in Beijing City. This work may provide guidance for setting safe limit for radon in drinking water in China in the future.

## 2. Experimental Section

### 2.1. Water Sampling

**Figure 1:**
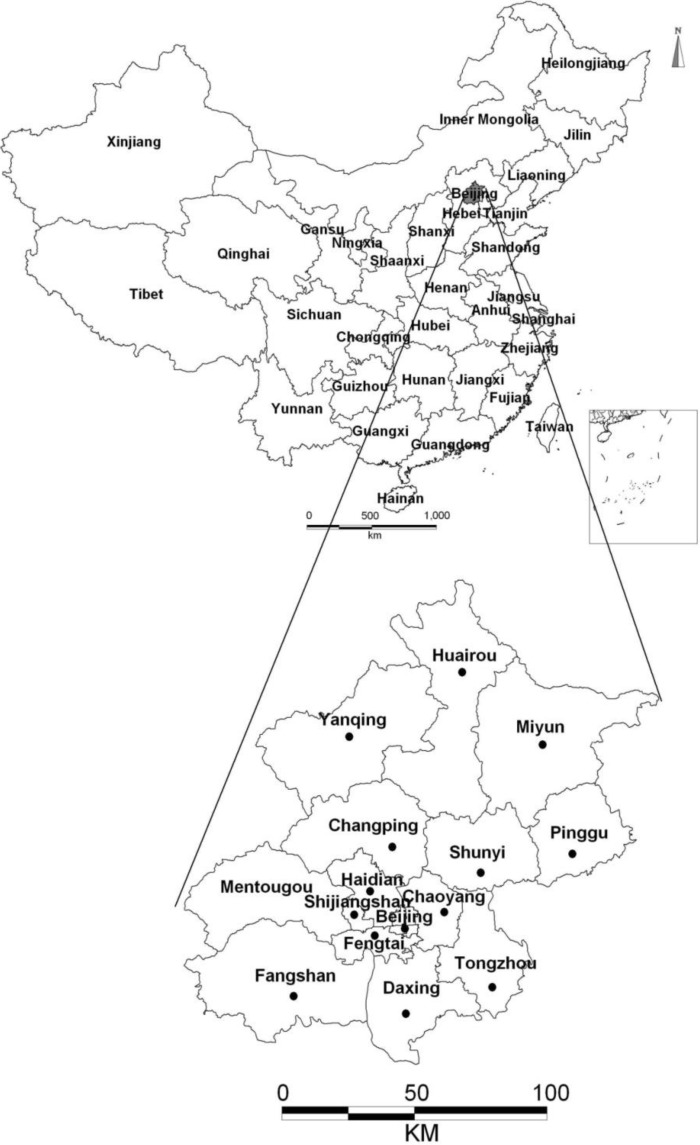
Location of Beijing City. The insets are zooms of the city map where the dots represent the sampling locations.

Beijing City, located in North of China, is the capital of China, and has a population of 21 million. The drinking water sources in the sampling area are groundwater and surface water, accounting for 40% and 60%, respectively. The water samples were collected from domestic water taps from public water supply or directly tapped by wells in the city and surroundings shown on the Figure 1. The samples were collected using 250 mL glass bottles during March, April and May of 2014. Before sampling, the water flowed for several minutes in order to collect the fresh water samples. The sample is filled carefully into the bottle from the bottom up without much turbulence, letting the water sample overflow. At this stage, gas leaks should be minimized by allowing the water to overflow sufficiently and capping the bottle without any air inside. The collected samples were transported to the laboratory to determine the radon concentrations with the minimum delay and were analyzed at the earliest possible time.

### 2.2. Method of Measurements

**Figure 2:**
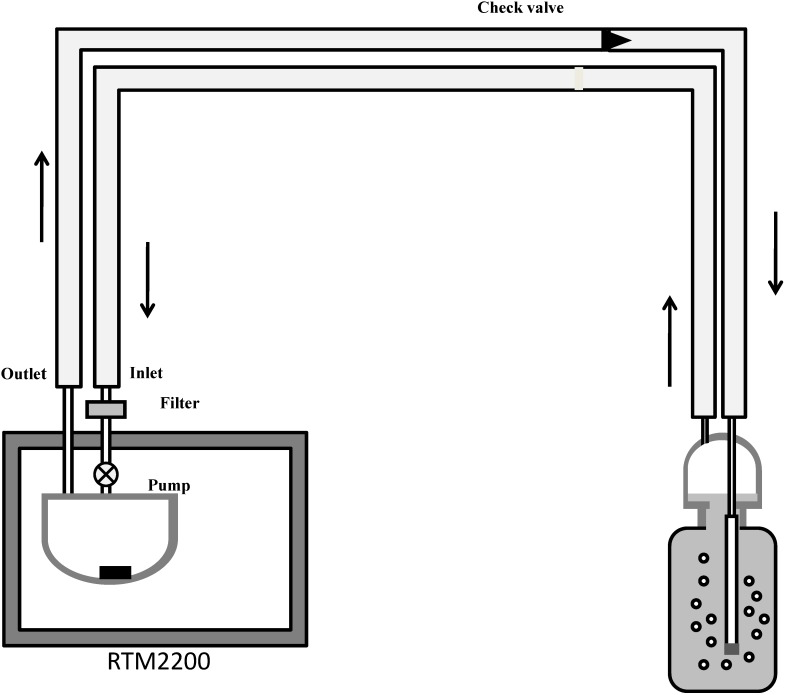
The set-up for radon measurements in water samples.

The analysis of radon in water is based on a simple method for the continuous monitoring of radon using a radon-in-air monitor coupled to an air-water exchanger. Figure 2 shows the set-up for radon measurements in water samples. A sample bottle with aerator is connected to a radon monitor RTM2200 (SARAD GmbH, Dresden, Germany) in a closed air-loop mode. The internal air pump in the RTM2200 re-circulates the air at a flow rate of about 0.25 L/min, purging radon in the water to achieve a rapid equilibrium of radon between water and air. Then, the activity of the radon is determined by counting its alpha-emitting daughters in the monitor. RTM2200 has an advantage that the detection efficiency cannot be influenced by moisture, so no desiccant acrylic column is needed.

In the RTM2200, the electrical field forces on the surface of a semiconductor detector collected the positively charged polonium daughters, ^218^Po^+^ (t_1/2_ = 33.05 min; alpha-energy = 6.00 MeV) and ^214^Po^+^ (t_1/2_ = 164 μs; alpha-energy = 7.67 MeV) at ground potential. The RTM2200 offers two calculation modes for the radon concentration, one (Slow) includes both ^218^Po and ^214^Po decays, and the other (Fast) includes ^218^Po only. The RTM2200 offers a high sensitivity of more than 3 cpm/(kBq/m^3^) (Fast Mode) obtained from a very small internal volume of only 250 mL. RTM2200 is set to run at a 5 min counting cycle, after 10 min, ^218^Po will almost reach equilibrium with ^222^Rn. Next the RTM2200 runs four counting cycles of five minutes each. Thus, the RTM2200 completes the sample measurement in 30 min.

The radon activity concentration equilibrium between water and air is determined by the respective partition coefficient, which depends on the temperature in the system as given in Equation (1) [14]:

*k* = 0.405*e*^−0.0502*T*^ + 0.105 (1)

where k is the ^222^Rn concentration ratio of water to air, and T is the temperature of the water in °C.

The actual activity of ^222^Rn in a water sample is the sum of ^222^Rn activities in the air loop and water in the sample bottle, where ^222^Rn is partitioned by Weigel’s Equation (2):

C_water_V_water_ = C_air_V_air_ + kC_air_V_water_ (2)

Therefore, the original activity of radon in the water sample, C_water_ (Bq/L), can be represented as:

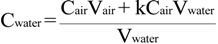
(3)
where C_air_ (Bq/L) is the activity of ^222^Rn in the air loop (after air-water equilibrium is achieved), V_water_ and V_air_ are the water volume and the air volume in the loop (detection chamber and tubes), respectively, and k is the distribution factor in Equation (1). The software SARAD radon in water calculator is used to calculate radon concentrations in water. The lower detection limit (LLD) for radon-in-water is about 0.268 Bq/L at confidence level of 2σ at a temperature of 25 °C.

In order to avoid contamination of the system, once a sample is measured, the monitor is flushed by fresh air for at least 15 min before a new sample, and the background is quite low so that it could be neglected for the analysis of radon in water.

## 3. Results and Discussion

### 3.1. Radon Concentrations

A total of 89 water samples from different locations were sampled and analyzed for their ^222^Rn content. Table 1 showed the radon concentrations for various water types. The means of radon concentrations for public water and well water were 4.63 ± 4.75 Bq/L and 11.41 ± 11.00 Bq/L, respectively. The results are much lower than those obtained for water samples from Baoji and Xianyang in Shanxi Province, China. The reported values for public water and well water were 12.36 and 38.85 Bq/L in Baoji, and those of Xianyang were 11.28 and 28.84 Bq/L [15].

**Table 1:** Radon concentrations for various water types.

Water Type	N	Mean(Bq/L)	Standard Deviation	Range(Bq/L)
Public water	73	4.63	4.75	LLD-29.00
Well	16	11.41	11.00	1.45-49.00

**Figure 3:**
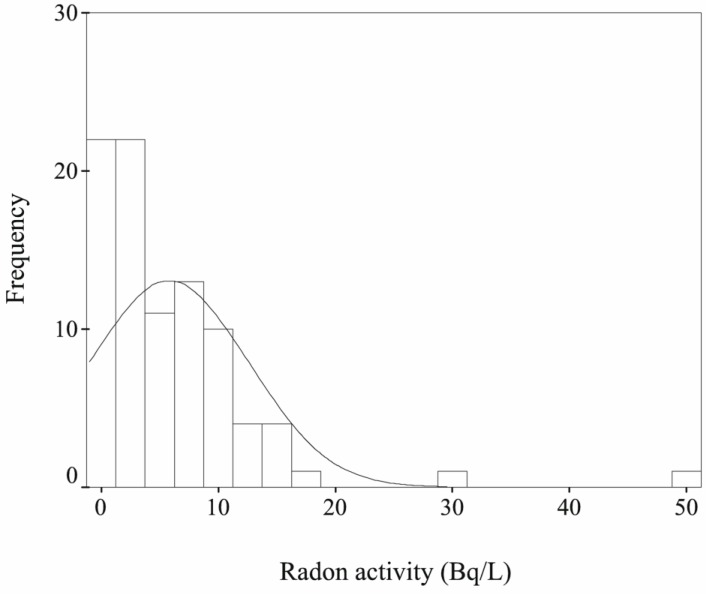
Frequency distribution of ^222^Rn activity in drinking water of Beijing City.

The distribution of radon concentrations for all measured water samples is shown in Figure 3 and it well fits to typical log-normal distributions. The observed radon levels range from detection limit up to 49 Bq/L. The calculated arithmetic and geometric means of radon concentrations in all measured water samples were equal to 5.87 and 4.63 Bq/L, respectively. These results are lower than the value of 15.46 Bq/L for radon in drinking water in Beijing City reported by Chen *et al.* in 1994 [16].The reason for this is that the overwhelming majority of public water source in Beijing changed from groundwater to mixture of groundwater and surface water in order to decline the hardness of drinking water since 2000.

With regard to the mean concentrations, the maximum radon concentration of 49 Bq/L was found in the water sample from the well located in Haidian area, and radon concentration of 29 Bq/L was found in water from public water supply in Miyun area, whereas the minimum value below the detection limit was found in water from surface water source. Groundwater accounts for 60% of the drinking water in Beijing City. Beijing locates in the central of North China, and faulted zones are prevalent. Lithology is more complex in Beijing. The rocks are mainly igneous rocks, metamorphic rocks, and sedimentary rocks. It is observed that the higher radon activity concentrations in groundwater generally correspond to that area where active fault zones lie in this study.

Twelve out of all sampled sites gave radon values of more than 11.11 Bq/L recommended by the EPA [3]. However, when compared with the European Union recommended level of 100 Bq/L for radon in public or commercial waters [6], all the analyzed samples from Beijing revealed radon values below the safe limit. The values obtained here are compared with those of reported in the literature from other countries, shown in Table 2. The concentrations of radon are generally higher than those observed in Poland, Mexico and India.

**Table 2:** Radon concentrations in various types of waters from other countries.

Water Type	N	Mean(Bq/L)	Range(Bq/L)	Country	Reference
Groundwater	260	2.63	0.9–5.1	India	[9]
Well	70	-	Background–3.8	Mexico	[10]
Well	27	9.28	1.42–53.64	Turkey	[11]
Tap	19	5.65	0.91–12.58	Turkey	[11]
Groundwater	89	1.92	0.42–10.52	Poland	[12]
Surface water	1511	15.4	0.5–129.3	Romaina	[13]
Groundwater					
Well					
Spring water					

To test the radon loss by water treatment and transport within run of water from water supply to the consumer, samples were taken at different locations, before and after water treatment and consumers’ tap. The radon activity concentration for several water supplies (WS) in Beijing City is presented in Table 3.

It is found that the radon activity concentration remains stable in some surveyed water units from the water source to the consumers. The main reason is that fresh water samples were collected letting the water flowed for several minutes before sampling and reduction of radon activity concentration within the run of the water from water supply to the consumer can be neglected. Furthermore, water treatment process for groundwater is simple, so before and after treatment radon activity concentration nearly same. However, aeration decreased radon activity, but not as effectively as expected, and dilution by mixing water with high radon activity concentrations with water with low radon activity concentration is more effective. These facts were also verified by Gruber *et al.* [17].

As for private wells, no water treatment process is conducted, except disinfection and the radon activity concentrations from well to the consumers remain close, so radiation protection attention has to be set to consumers who directly drink water of the wells, where the highest radon activity concentrations were measured within this survey.

**Table 3:** Radon concentrations for samples taken at different locations in water supplies.

No.	Water Type	Before Treatment (Bq/L)	After Treatment (Bq/L)	Consumers’ Tap (Bq/L)
WS_1_	Public water	32.63 ± 1.75	28.53 ± 2.23	29.00 ± 0.22
WS_2_	Public water	12.19 ± 0.57	7.87 ± 1.12	9.50 ± 1.12
WS_3_	Public water	12.39 ± 0.93	11.23 ± 0.66	11.37 ± 1.23
WS_4_	Public water	7.59 ± 1.36	3.56 ± 0.86 ^a^	3.68 ± 0.81
WS_5_	Public water	15.68 ± 1.52	3.56 ± 0.46 ^b^	-
WS_6_	Public water	11.72 ± 1.61	10.80 ± 1.33	-
WS_7_	Public water	11.72 ± 1.61	11.12 ± 0.87	-
WS_8_	Public water	13.19 ± 1.32	11.12 ± 0.87	-
WS_9_	Public water	19.44 ± 1.11	19.53 ± 1.38	-
WS_10_	Public water	14.45 ± 1.85	4.61 ± 0.36 ^b^	-
WS_11_	Public water	16.58 ± 0.54	4.56 ± 0.42 ^b^	-
WS_12_	Public water	18.19 ± 2.28	5.59 ± 0.93 ^b^	-
WS_13_	Public water	26.19 ± 1.60	24.98 ± 1.37	-
WS_14_	Well	14.71 ± 0.97	-	14.69 ± 0.39
WS_15_	Well	7.48 ± 0.43	-	8.23 ± 1.15
WS_16_	Well	14.28 ± 0.82	-	17.94 ± 0.67
WS_17_	Well	49.00 ±2.84	-	52.85 ± 2.08

Notes: -: Means it hasn’t been measured; **^a^**: after aeration. **^b^**: after dilution with water with low radon activity concentration.

### 3.2. Effective Dose Calculation

The total annual effective dose for general population caused by occurrence of radon in drinking water and its domestic use is a sum of the effective doses due to radon ingestion with water and inhalation from waterborne radon. The annual effective dose due to intake of radon from drinking water can be calculated from Equation (4) [18]:

*E_ing_* = *DCF* × *A* × *V* (4)

where DCF (dose conversion factor) or dose coefficient is in Sv/Bq, A is average radon activity in drinking water in Bq/L, V is annual volume of water consumed directly from tap in L.

The estimated dose coefficient due to ingestion of radon from water is 10^−8^ Sv/Bq for an adult [19]. Since radon is readily lost from water by heating or boiling, the value of 60 L for the weighted direct annual consumption of tap water has been proposed in United Nations Scientific Committee on the Effects of Atomic Radiation (UNSCEAR) 2000 Report [20].

Therefore, for the average radon concentration of 4.63 Bq/L the effective dose from water ingestion will be 2.78 μSv and for maximal observed radon concentration in water of 49 Bq/L corresponding dose is 29.40 μSv. These doses in comparison with average effective dose from all natural sources 2.4 mSv are really negligible.

The dose from inhalation of water-borne radon can be calculated from following Equation (5):

*E_inh_* = *DCF* × *A* × *T* × *F* × *t* (5)

where DCF is a radon dose conversion factor for radon inhalation DCF = 22×10^−9^ (Sv/(Bq∙h∙m^−3^), A is the average radon concentration in Bq/L, T is the radon transfer from water to air coefficient T = 0.1 L/m^3^. t is the average annual indoor occupancy in hours t = 7000 h. F is the indoor radon—daughters equilibrium factor F = 0.4.

Introducing the above described values, the annual effective dose for average radon content in water is 28.5 μSv and for maximal observed radon concentration in water corresponding dose is 301.84 μSv.

These doses due to inhalation of water-borne radon are one order higher of those from radon ingestion with water. They are comparable and even higher than that of the annual effective dose received by the southern Poland population [12]. Therefore, it could be concluded that not the ingestion of waterborne radon but inhalation of the radon escaping from water is substantial part of radiological hazard.

## 4. Conclusions

Radon concentrations have been determined from 89 drinking water samples collected from domestic water taps from public water and wells around Beijing City. The maximum radon concentration of 49 Bq/L was found in a water sample directly extracted from a well in this work. Twelve out of all sampled sites gave radon values of more than the 11.11 Bq/L recommended by the EPA. All the analyzed samples from Beijing revealed radon values below 100 Bq/L the reference level recommended by the European Union. At present there is no reference level for radon in drinking water in China. The results of this study may provide guidance for setting safe limit for radon in drinking water in China in the future.

As for radon reduction in water, water treatment processors have little effect on radon reduction in some water units surveyed from the water source to the consumers. However, aeration decreased radon activity, and dilution by mixing water with high radon activity concentrations with water with low radon activity concentration is more effective.

The annual effective dose due to inhalation of water-borne radon is one order higher of those from radon ingestion with water. It could be concluded that not the ingestion of waterborne radon but inhalation of the radon escaping from water is substantial part of radiological hazard. In general, radon in water is a big concern for public health, especially for consumers who directly use well water with very high radon concentration in household.
